# Dlx1 and Rgs5 in the Ductus Arteriosus: Vessel-Specific Genes Identified by Transcriptional Profiling of Laser-Capture Microdissected Endothelial and Smooth Muscle Cells

**DOI:** 10.1371/journal.pone.0086892

**Published:** 2014-01-28

**Authors:** Regina Bökenkamp, Ronald van Brempt, Jacoba Cornelia van Munsteren, Ilse van den Wijngaert, Ronald de Hoogt, Livio Finos, Jelle Goeman, Adriana Cornelia Gittenberger-de Groot, Robert Eugen Poelmann, Nicolaas Andreas Blom, Marcus Cornelis DeRuiter

**Affiliations:** 1 Department of Pediatric Cardiology, Leiden University Medical Center, Leiden, The Netherlands; 2 Department of Intensive Care, Leiden University Medical Center, Leiden, The Netherlands; 3 Johnson and Johnson Pharmaceutical Research and Development, Beerse, Belgium; 4 Department of Anatomy and Embryology, Leiden University Medical Center, Leiden, The Netherlands; 5 Department of Medical Statistics and Bioinformatics, Leiden University Medical Center, Leiden, The Netherlands; 6 Biostatistics, Department for Health Evidence, Radboud University Medical Center, Nimegen, The Netherlands; 7 Department of Cardiology, Leiden University Medical Center, Leiden, The Netherlands; Cincinnati Children’s Hospital Medical Center, United States of America

## Abstract

Closure of the ductus arteriosus (DA) is a crucial step in the transition from fetal to postnatal life. Patent DA is one of the most common cardiovascular anomalies in children with significant clinical consequences especially in premature infants. We aimed to identify genes that specify the DA in the fetus and differentiate it from the aorta. Comparative microarray analysis of laser-captured microdissected endothelial (ECs) and vascular smooth muscle cells (SMCs) from the DA and aorta of fetal rats (embryonic day 18 and 21) identified vessel-specific transcriptional profiles. We found a strong age-dependency of gene expression. Among the genes that were upregulated in the DA the regulator of the G-protein coupled receptor 5 (*Rgs5*) and the transcription factor distal-less homeobox 1 (*Dlx1)* exhibited the highest and most significant level of differential expression. The aorta showed a significant preferential expression of the Purkinje cell protein 4 (*Pcp4*) gene. The results of the microarray analysis were validated by real-time quantitative PCR and immunohistochemistry. Our study confirms vessel-specific transcriptional profiles in ECs and SMCs of rat DA and aorta. *Rgs5* and *Dlx1* represent novel molecular targets for the regulation of DA maturation and closure.

## Introduction

The ductus arteriosus (DA) is a highly specialized fetal blood vessel that connects the pulmonary trunk with the descending aorta. In the fetal circulation the DA bypasses the fluid-filled lungs by shunting blood from the pulmonary trunk into the systemic circulation. At birth, with the transition from placental oxygen supply to air breathing, this connection becomes unnecessary and closes. DA closure is a crucial step in the transitional circulation as it ensures the efficient delivery of oxygenated blood from the lungs to all organs. Failure of DA closure leads to one of the most common cardiovascular anomalies in children causing morbidity and mortality especially in premature infants. [1], [2] Inhibition of DA closure on the other hand is critical for the immediate postnatal survival of infants with various types of severe structural heart disease. [3] Although sharing the same embryologic origin and environment the DA shows a unique development and differentiation compared with other pharyngeal arch artery derivatives in rats and many other species. [4], [5] In the chick embryo the DA acquires its typical muscular morphology while the adjoining aorta differentiates into an elastic vessel by elastin deposition and concomitant loss of actin expression in the media. [5] Already in the second trimester of human gestation the DA media is composed of highly differentiated vascular smooth muscle cells (SMCs) [6] compared to the aorta. From the second trimester of pregnancy onwards endothelial cells (ECs) and SMCs in the inner part of the DA undergo a ductus-specific remodeling process. [7] As a result of this process the mature DA exhibits intimal thickening, which facilitates sealing of the constricting vessel immediately after birth. Early (functional) closure of the DA is triggered by the postnatal increase of oxygen partial pressure and decrease in prostaglandin. [8] The contraction in response to oxygen is intrinsic to the SMCs of the DA and highly conserved among different species. Oxygen-induced inhibition of voltage-gated K^+^ channels, activation of L-type voltage-gated calcium channels, increased endothelin synthesis and redox-dependent activation of ROCK [9] and other calcium sensitization pathways [10] have been documented in the DA of humans and rabbits. After the functional closure the definitive anatomical closure of the DA is initiated. In the subsequent postnatal period the DA degenerates due to cytolytic necrosis and apoptosis of the muscular artery into a fibrous remnant, [11] while the adjoining elastic vessels remain open.

Although various anatomical, physiological and biochemical aspects of DA closure have been studied during the last decades reviewed in Bökenkamp et al. [8] little is known about the molecular mechanisms controlling the unique remodeling process of the DA. A recent study documented the expression of the truncated lamin A protein “progerin”, the underlying cause of Hutchinson Gilford Progeria syndrome, related to the cytolytic necrosis process in the DA. This finding suggests involvement of alternative splicing of lamin A (*LMNA*) in the vascular remodeling of the normal neonatal DA [12].

Human disease states offer insights into the transcriptional regulation of certain physiological processes. Regarding DA closure Char syndrome and patent DA in preterm infants are instructive. The patent DA and mild facial and hand anomalies in Char syndrome are caused by mutations in the neural crest-related transcription factor AP2B gene (*TFAP2B*). [13] Sequence polymorphisms in the same gene are associated with isolated non-syndromic patent DA in preterm infants [14]–[16] and consequently suggest a role for *Tfap2B* in the transcriptional control of normal DA closure. Targeted deletions of *Tfap2B* in mice confirm the critical role of *Tfap2B* in DA development and limb patterning [17], [18].

Two previous studies on transcriptional profiles of the DA and aorta in fetal rats revealed characteristic differences between both vessels. [19], [20] These studies are hardly comparable [21] as one was aimed to ascertain changes in in aorta and DA linked to birth and oxygen action [19] and the other addressed the issue of the relative predominance of transcripts in the DA versus the aorta antenatally. [20] Upregulation of sarcomeric genes, characteristic for the fetal DA in the study of Costa et al. [19] was found in the aorta by Jin et al. [20]. Growth hormone receptor (*Ghr*) exhibited the most significant upregulation in the DA among all DA-specific genes in the study of Jin et al. [20] but was not differentially expressed in the dataset of Costa et al [19.] Both studies used pooled whole vessel preparations of DA and aorta from two different strains of rats (i.e.,Wistar [19] and Long-Evans [20]) for the microarray analysis.

In microarray analyses of whole vessel extracts the specific role of different cell populations, *i.e*., ECs and SMCs of the DA and descending aorta, cannot be determined. We have chosen a more selective technique. Laser-captured microdissected cells offer the opportunity to study gene expression profiles in neighboring cells of different embryonic origin. By analyzing the profiles from late fetal (day 18) and near term (day 21) rats we were able to study the change in ECs and SMCs during the DA remodeling process at late gestation. We hypothesized that both cell types of the DA and aorta would have distinct transcriptional profile characterizing the artery that will close after birth and the neighboring vessel that will remain open for the duration of life. We further hypothesized that transcriptional profiles in ECs and SMCs from DA and aorta would reflect either a different origin or function of these cells, which might be detected in the perinatal period accompanying the radical changes of environment and phenotype.

## Methods

### 1. Animals

**Figure 1:**
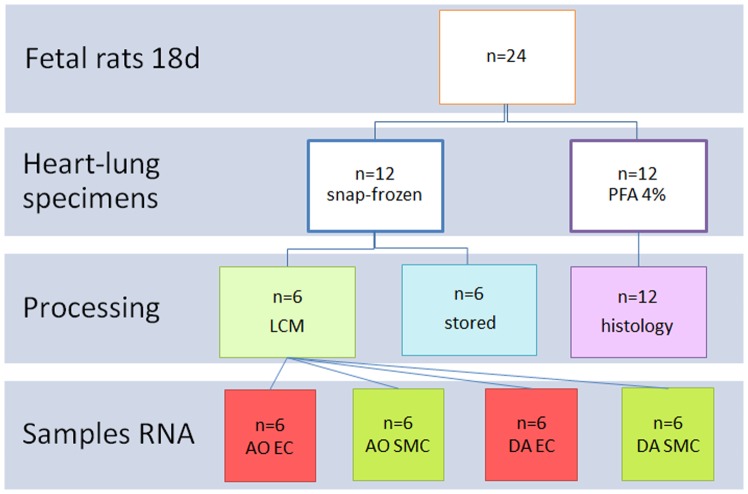
Flowchart showing the experimental design for fetal rats at day 18. The fetal rats were delivered from 2 dams. LCM =  laser-capture microdissection, AO EC = endothelial cells from the descending aorta, AO SMC = smooth muscle cells from the descending aorta, DA EC = endothelial cells from the ductus arteriosus, DA SMC = smooth muscle cells from the ductus arteriosus, PFA 4% = paraformaldehyde 4%, LCM = laser-capture microdissection, MA = microarray. The same experimental design was used for the experiments at day 21.

Pups from 6 timed–pregnant Wistar rats were delivered after respectively 18 and 21 days of gestation (spontaneous birth occurs at 22 days of gestation) by hysterotomy through a median abdominal incision. For the time of delivery the dams were anesthesized with sevofluorane 2.5% in room air; subsequently the dams were euthanized. Care was taken to keep the time between the delivery of the first and the last pup within 5 minutes. The litter size was approximately 12 pups per dam. Out of these 12 the first 3 pups were used for the microarray experiment. Isolation of the fetal thorax was carefully performed in RNase free conditions, frozen in Tissue Tek (Sakura Finetek USA, Torrance, CA, USA), and stored at −80°C. Figure 1 shows the experimental design, which was used at both day 18 and day 21 of gestation. The feasibility of the experiments was tested in a separate pilot experiment using 4 Wistar rat embryos from two dams after 21 days gestation. Data from the pilot experiment are presented in Figures S1 and S2.

### 2. Ethics Statement

Both experiments were approved by an independent Institutional Animal Care and Use committee at the LUMC in accordance with the Helsinki convention for the use and care of laboratory animals.

### 3. Laser-capture Microdissection

**Figure 2:**
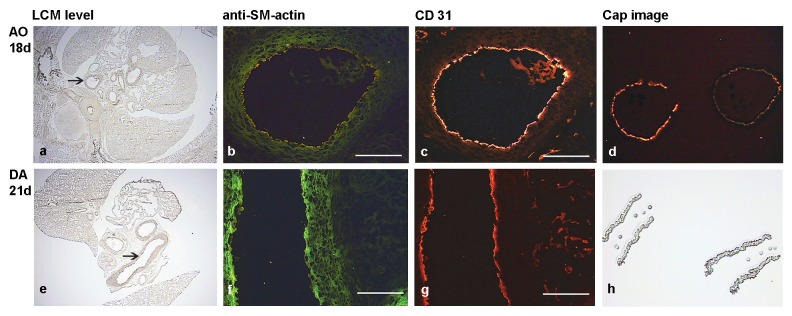
Sections used for laser-capture microdissection. The level of laser-capture microdissection (LCM level) is indicated in **a** and **d**. The arrow represents the area of LCM in the aorta (AO) of a fetal rat at 18 days (18 d) and the ductus arteriosus (DA) at 21 days (21 d). Smooth muscle cells are labeled with anti-smooth muscle actin in green (**b**, **f**) and endothelial cells are labeled with CD31 in red (**c,g**). Bars represent 100 µm. Images from the microdissection cap show the dissected endothelium of two aorta sections with fluorescence (**d**) and strips of SMCs from the DA without fluorescence (**h**). These photomicrographs are taken from the microdissection cap that was used for capturing the tissue samples from the sections.

Cryostat sections (8 µm) were attached to RNase free (Superfrost ™) microscopic slides and immediately placed on dry ice. The slides were preserved in −80°C conditions. Three slides of each embryo were immunohistochemically stained in one batch. RNase free PBS was used for the appropriate dilution of antibodies and washing steps. Furthermore, Superase. In (AM2696, Ambion, Austin, TX, USA) was added to the diluted antibody solution (1 U/µL). After fixation in cold acetone (4°C) for 2 minutes, cold (4°C) conditions were maintained during further immunohistochemical staining. The three slides could easily be air-dried after acetone fixation. 30 µL of cold PBS was applied to each tissue section and drained off. In subsequent order, 30 µL of Biotin-conjugated mouse anti-rat CD31 (BD 555026) (5 µg/100 ul), Cy3 Streptavidin (1∶100) Streptavidin Cy3 (HistoGene® Cy3® streptavidin) taken from Histogene® LCM Immunofluorescence Staining Kit KIT0420(Applied Biosystems, Foster City CA, USA) and alpha smooth muscle actin antibody (1A4) FITC (Abcam ab8211) (1 µg/30 µL) were applied. In order to further limit RNase activity, each antibody solution was only applied for 2 minutes. Subsequently, the antibody was gently drained off with 200 µL of cold PBS. The slides were dehydrated at room temperature in 75% EtOH (30 sec), 95% EtOH (30 sec), 100% EtOH (30 sec), 100% EtOH (120 sec), xylene (180 sec). Dehydration was immediately followed by laser capture microdissection (LCM) (Veritas Microdissection Instrument, Arcturus Bioscience Inc., Mountain View, CA, USA). 100 to 200 cells from three tissue sections were dissected from the DA and descending aorta of each embryo. ECs were defined as cells lining the lumen and staining positive for anti-rat CD31. Alpha-smooth muscle actin positive cells were identified as SMCs. (Figure 2) The microdissected cells were transferred to a Gene Amp tube (Applied Biosystems, Foster City CA, USA) containing 75 µL of Rneasy lysis buffer (RLT; Qiagen, Hilden, Germany) containing 0.14 M beta-mercaptoethanol and 200 ng Polyinosinic Acid (Sigma Aldrich, USA). The samples were incubated at 42°C for20 minutes and stored at −80°C.

### 4. RNA Isolation, Amplification, Labeling, and Microarray Hybridization

The 48 laser-captured samples were processed simultaneously after thawing. From each of the 6 parallel samples (Figure 1) RNA was isolated, cleaned up and eluted separately in 14 µL of RNase-free water according to the RNeasy Minelute protocol (Qiagen, Hilden, Germany). Two rounds of linear mRNA amplification were performed (modified Eberwine protocol): two-cycle cDNA synthesis and synthesis of biotin-labeled aRNA was performed according to the GeneChip Eukaryotic Sample and Array Processing Manual (Affymetrix, Santa Clara, CA, USA). MEGAscript T7 kit (Ambion, Austin, TX, USA) was used for in vitro transcription of the second cDNA strand in the first round of amplification. The second round of amplification, starting from 100 ng of first round aRNA, was finalised during the GeneChip in vitro transcription (IVT) labelling. Despite immunohistochemic double-staining and laser-capture dissection sufficient amounts of RNA could be isolated from these cells and amplified for the microarray experiment. (see Table S1) Two-round linear mRNA amplification yield). Labeled RNA was hybridized to the GeneChip Rat Genome 230 2.0 Array. Hybridization was performed using 12.5 µg of biotin-labeled RNA at 45°C for 16 hours under continuous rotation. Arrays were stained in Affymetrix Fluidics stations using streptavidin-phycoerythrin (SAPE), followed by staining with anti-streptavidin antibody and a second SAPE staining. Subsequently, arrays were scanned with an Agilent Laserscanner (Affymetrix, Santa Clara, CA, USA).

### 5. Real Time Quantitative PCR Analysis

First-strand cDNA synthesis was performed on 1 µg of the 2^rnd^ linear amplification cRNA from the microdissected cells using random hexamer primers and Superscript III RT (Invitrogen, Grand Island, NY, USA) in a total reaction volume of 20 µL during 1 h at 50°C. This was followed by inactivation of the enzyme at 70°C for 15 min (Invitrogen, Carlsbad, USA). Quantitative PCR (qPCR) was performed on an ABI Prism 7900-HT Sequence Detection System (Applied Biosystems, Carlsbad, USA). The total PCR reaction volume was 10 µL. The reaction mix was prepared using a qPCR Core kit (Eurogentec RT-QP73-05). The concentrations of each dNTP and MgCl_2_ were 200 µM and 5 mM respectively. A final dilution of 300 nM of the forward and reverse primer and 100 nM dilution of the probe of each assay were utilized in the reaction mix. 10 ng cDNA of each sample was used in the PCR reaction. 20 ng of whole embryonic rat cDNA was serially diluted (1X, 4X, 16X, 64X, 256X, 1024X) for the construction of a standard curve for each assay. A duplicate of each PCR reaction was performed. The thermal cycling conditions were 10 min at 95°C, followed by 45 cycles of 15 s at 95°C and 1 min at 60°C. Validated pre-designed Taqman Gene Expression Assays (Applied Biosystems, Carlsbad, USA) corresponding to the housekeeping genes *Pgk1* (Rn00821429_g1) and *Ppib* (Rn00574762_m1) were used to generate standard curves on serial dilutions of cDNA. The standard curve method was used to calculate the relative quantity of each gene expressed in the tissue samples. Relative expression values of *Dlx1*, *Rgs5*, *Pcp4*, *vWF* and *Tfap2B* were normalized with housekeeping genes *Pgk1* and *Ppib*. Linear regression (least squares) and robust regression were applied for the construction of calibration curves. Robust regression [22) gives less weight to large observations (for high variant replicates). GraphPad Prism 5 (GraphPad Software, Inc., La Jolla, USA) and the lmrob function in the ‘Robustbase Package’ in R (http://www.inside-r.org/packages/cran/robustbase/docs/.vcov.avar1) were used for visualization and statistical analysis. In the figures data are presented as means with standard errors of the mean (SEM). Details of each assay, Y-intercept and slope (linear and robust regression) and calibration curve (robust regression) are provided in Table S2. This table contains also the graphs presenting the Ct values of the samples along the calibration curve (robust regression) for each assay.

### 6. Statistical Analysis

The Affymetrix probe level data were summarized using FARMS (Factor Analysis for Robust Microarray Summarization). Raw intensities were log2 transformed to get data normally distributed. First, an unsupervised multivariate projection method, Spectral Map Analysis, was applied to reduce the complexity of highly dimensional data (n genes versus p samples). Spectral Map Analysis provides an unbiased identification of the predominant clusters of genes and subjects that are present in the data set. Second, we modeled the expression data in LIMMA (Linear Models for Microarray Data) [23), a method that is designed to get reliable analyses even for experiments with small number of arrays. In LIMMA, we used a model that includes the three dichotomous factors of cellular origin (anti-CD31 cells versus anti-alpha smooth muscle actin cells), tissue origin (aorta versus DA), and time (day 18 versus day 21), as well as all two-way interactions between these factors. In this model, we performed tests for differential gene expression between the two cellular origins. Third, differences between DA and aorta in expression profiles over embryonic age were investigated by testing for the presence of a two-way interaction of tissue origin and time, using the same model in LIMMA. Models like LIMMA assume that all the samples have been randomly and independently collected. In all our LIMMA analyses, we tested for the differences corresponding to each factor both globally over all samples to obtain increased power, but also separately for subsets of the samples corresponding to specific values of the other factors, to be able to detect whether effects are consistent for different values of the other factors. We corrected each of the analyses separately for multiple testing, using the false discovery rate (FDR) and corrected all p-values for multiplicity. Multiplicity-adjusted p-values below 0.05 were considered significant. The complete set of data is available in the Gene Expression Omnibus (http://www.ncbi.nlm.nih.gov/geo/query/acc.cgi?acc=GSE51248).

### 7. Immunohistochemistry (see Figure S3)

Immunohistochemistry was used as a qualitative technique to confirm protein expression in tissue sections. By rigorously standardizing the methodology (simultaneous staining of sections using the same aliquots, timing and temperature) we were able to validate differences in staining intensity between sections, albeit not the degree of difference.

The experiments were performed on sections from 4% paraformaldehyde fixed tissue routinely processed for paraffin immunohistochemical investigation. Primary antibodies against Rgs5 (Santa Cruz Biotechnology, CA, USA, SC-28492) 1∶200–1∶400, Pcp4 (Santa Cruz Biotechnology,CA, USA, SC–98549) 1∶200–1∶400, DLX-1 (Aviva Systems Biology, San Diego, CA, ARP32866) 1∶1000, Tcfap2B (Abcam, Cambridge, MA, USA ab18113) 1∶200 were used. The primary antibodies were dissolved in phosphate buffered saline (PBS) with 0.05% Tween-20 and 1% bovine serum albumin (BSA, Sigma Aldrich, USA). Slides were incubated overnight. Between subsequent incubation steps all slides were rinsed two times in PBS and once in PBS/Tween-20. The slides were incubated with secondary antibodies for 45 min: for RGS 5 with biotinylated horse anti -goat (H+L) (Vector laboratories, USA BA–9500), 1∶200 and 1∶66 normal horse serum (Brunschwig Chemie, Switzerland, S-2000), for PCP4 and DLX1 with biotinylated goat anti-rabbit (H+L) (Vector Laboratories, USA, BA-1000), 1∶200, for TCFAP2B with biotinylated horse anti-mouse (H+L) (Vector Laboratories, USA, BA-2000), 1∶200 and 1∶66 normal horse serum (Brunschwig Chemie, Switzerland, S-2000). The slides were incubated with ABC-reagent (Vector Laboratories, USA, PK-6100) for 45 min. For visualization the slides were incubated with 400 µg/ml 3-3′di-aminobenzidintetrahydrochloride (DAB, Sigma-Aldrich Chemie, USA, D5637) dissolved in Tris-maleate buffer pH 7.6 to which 20 µlH_2_O_2_ was added for 8 minutes. Counterstaining was performed with 0.1% haematoxylin (Merck, Darmstadt, Germany) for 5 sec., followed by rinsing with tap water for 10 min. All slides were dehydrated and mounted with Entellan (Merck, Darmstadt, Germany).

## Results

**Figure 3:**
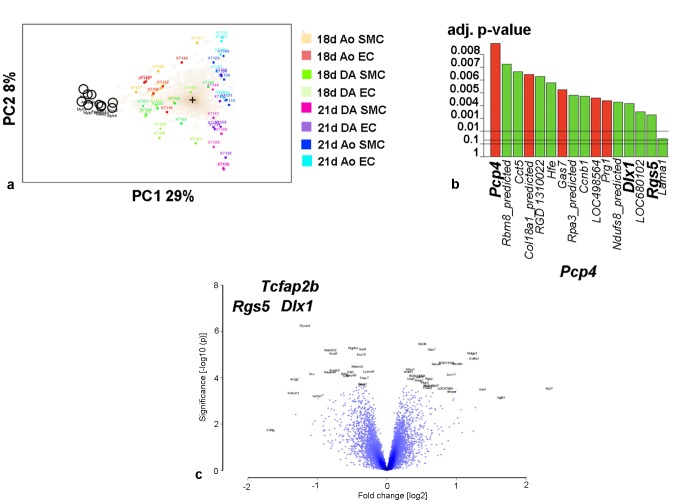
Visual representation of microarray results. A. Spectral map bioplot. The first two principal components (PC) of the weighted spectral map analysis (SPM) of normalized microarray data are plotted. The samples are depicted in coloured squares with numbers. The colours are explained in the figure. AO SMC = smooth muscle cells from the descending aorta, DA EC = endothelial cells from the ductus arteriosus, DA SMC = smooth muscle cells from the ductus arteriosus. 18 d = day 18 of gestation, 21 d = day 21 of gestation. Distances between the squares are a measure for the similarity between samples. Genes that do not contribute to the differences are indicated as dots in the cloud around the centroid (represented by the cross). The ten most significantly contributing genes are annotated by their gene symbol. The first PC (PC1) explains 29% of the variance in the dataset and discriminates samples from day 18 (n = 24) from those of day 21 (n = 24). The second PC (PC2) explains 8% of the variance and discriminates between ECs and SMCs. B. Histogram showing the most significant differentially expressed genes between DA and aorta. The annotation of the genes is on the x-axis. The adjusted p-values are on the y-axis. Red bars represent genes that are enriched in the aorta. Green bars represent genes that are upregulated in the DA. C. Volcano plot. The volcano plot constructed with LIMMA analysis summarizes the fold changes between the two types of the samples (*i.e.*, DA versus aorta) and the log10 transformed p-values. The negative log10 transformed p-values (y-axis) are plotted against the log ratios between the samples (log_2_ fold change). For our study we selected 4 genes. The position in the upper left (*Rgs5, Tfap2B, Dlx1*) is the result of a high ratio of differential expression.

First we explored the microarray data of all 48 samples by the unsupervised spectral map analysis (SPM). This analysis revealed differences between the 24 samples from day 18 and the 24 samples from day 21 (Figure 3a). The SPM bioplot illustrates the results of this unsupervised analysis. The first component of the principal component analysis explained 29% of the total variance in the dataset of the 11484 genes that were reliably detected, and separated the samples from day 18 from those from day 21. The second component explained 10% of the total variance and separated ECs from SMCs. Next we analyzed the dataset with a supervised test. With the linear model for microarray data analysis (LIMMA) (*P*-values <0,05 adjusted for multiple testing using the False Discovery Rate (FDR) we compared all samples by gestational age (*i.e.,* day 18 versus day 21), by cell type (*i.e.,* ECs versus SMCs) and by vascular origin (*i.e.,* DA versus aorta). LIMMA confirmed the strong age-dependency of the gene expression detected by the unsupervised test. In the gene-by–gene analysis 3239 genes were differentially expressed between the 24 samples from day 18 and the 24 samples from day 21. Compared to other factors such as cell type and vascular origin gestational age had the largest effect on the distribution of the data.

Comparing ECs and SMCs we found differential expression of 858 genes in the gene-by-gene analysis. Examples include well-known endothelial-specific genes such as tyrosine kinase with immunoglobulin-like and EGF-like domains 1 (*Tie1*), endothelial tyrosine kinase, (*Tek*), platelet/endothelial cell adhesion molecule 1 (*Pecam1*) and von Willebrand factor (*Vwf*). (see Figure S4) The latter was found to be the highest upregulated gene (5.1 logfold) in ECs (adjusted p-value 1.8e-06) in the LIMMA analysis. The upregulation of *Vwf* was also validated by rtqPCR confirming that the LCM procedure was successful for the enrichment of EC (see Figure S4).

The comparison between the DA and the aorta revealed 16 genes that were differentially expressed between both vessels (Figure 3b). Five out of these 16 genes were significantly upregulated in the aorta. Among these genes *Pcp4* showed the highest (3.2 log-fold, adj. p<0.001) and the most significant upregulation. Out of the 9 genes that were upregulated in the DA *Rgs5* (3.1 log-fold, adj. p = 0.045) had the highest expression level. Table 1 lists the 16 genes alphabetically. Volcano plots are provided (Figure 3c and Figure S5). The volcano plot (Figure 3c) shows the comparison between DA and aorta in all 48 samples. From this plot we selected four genes (*Rgs5*, *Dlx1* and *Tfap2B* in the DA and *Pcp4* in the aorta) for further study. These four genes were found in the left (*Pcp4*) or right (*Rgs5*, *Dlx1*, *Tfap2B*) upper part of the plot.

**Table 1:** The 16 most significantly differentially expressed genes between DA and aorta in alphabetical order.

Probe set ID	Gene Name (UniGene)	Symbol	LogFC	p -value
Affymetrix			DA:Ao	
25203_at	Cyclin B1	*Ccnb1*	0.587	<0.050
294864_at	Chaperonin containing TCP1	*Cct5*	0.509	<0.045
304021_at	Collagen type 8 alpha	*Col8a1*_predicted	−2.081	<0.001
296500_at	Distal-less homeobox1	*Dlx1*	1.741	<0.050
85246_at	Growth arrest specific protein 7	*Gas7*	−0.676	<0.021
29199_at	Hemochromatosis gene	*Hfe*	0.426	<0.050
316758_at	Laminin alpha 1	*Lama1*	1,42	<0.050
1383708_at	Integrin, beta-like protein 1 (Itgbl1)	*LOC498564*	−2.216	<0.050
1378988_at	mab-21-like 2	*LOC680102*	0.947	<0.050
293652_at	NADH dehydrogenase (ubiquinone) Fe-S protein 8	*Ndufs8*_predicted	0.573	<0.045
25510_at	Purkinje cell protein 4 (also known as neuron specific protein PEP-19)	*Pcp4*	−3.185	<0.001
295401_at	Lipidphosphate phosphatase-related protein type 4 (LPPR4)	*Prg1*	−0.753	<0.050
295284_at	RNA binding motif protein 8A	*Rbm8*_predicted	0.388	<0.050
1372066_at	Family with sequence similarity 103, member A1	*RGD1310022*	0.709	<0.045
54294_at	Regulator of G-protein signaling 5	*Rgs5*	3.075	<0.050
296883_at	Replication protein A3	*Rpa3*_predicted	0.642	<0.045

Probe set identification numbers (ID Affymetrix) of the GeneChip Rat Genome 230.20 Array, gene name according to UniGene, gene symbols, logfold changes (logFC) between DA and aorta and the adjusted p-values are shown.

### Rgs5

**Figure 4:**
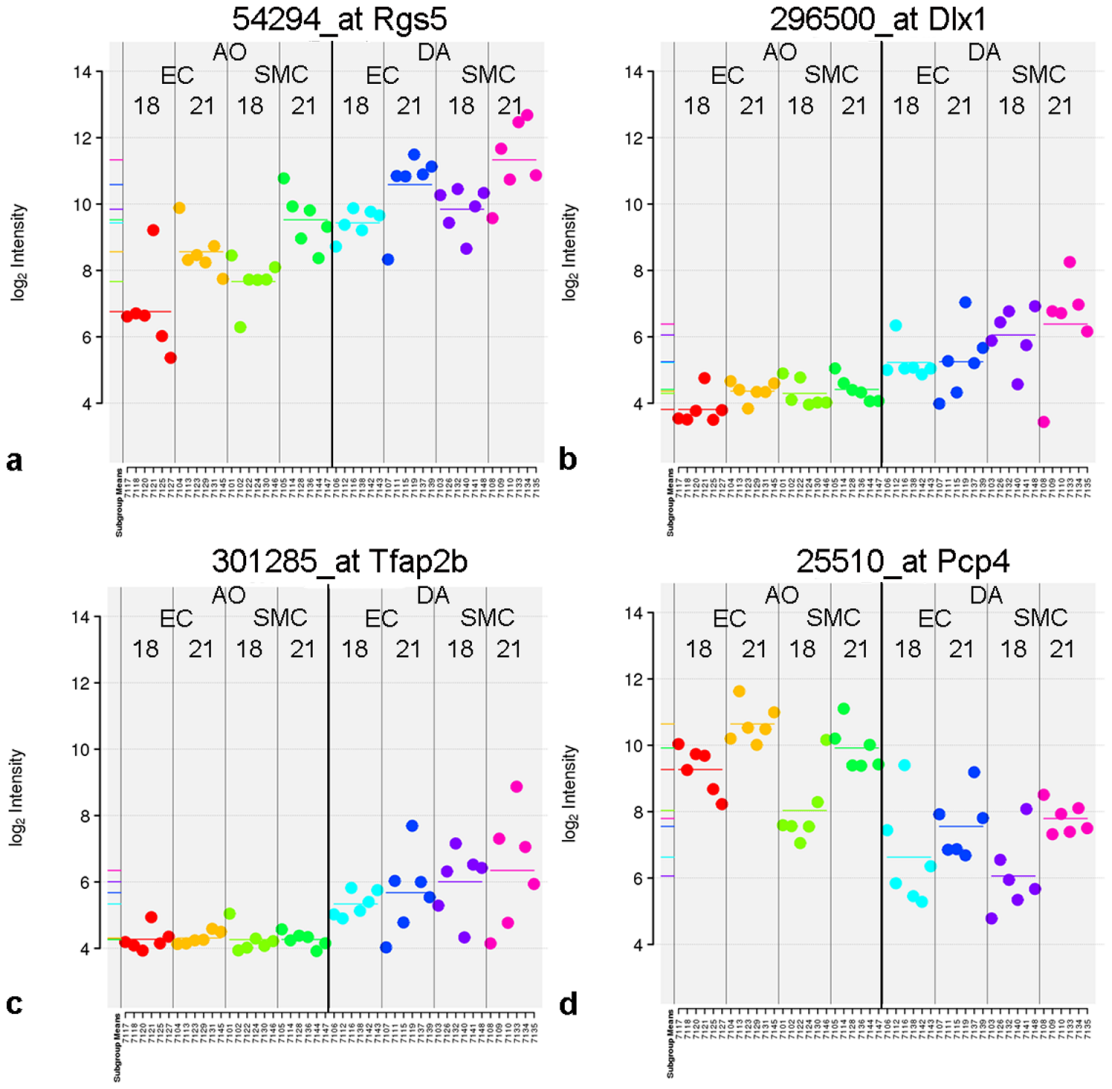
Gene expression of *Rgs5* (a), *Dlx1* (b), *Tfap2B* (c) and *Pcp4* (d) by microarray. Expression levels are expressed as fluorescent signal intensity measured on the array after normalization. The dots represent individual samples. Horizontal bars represent the means. The same colors are used in a–d. Red = ECs from the aorta at day 18 (AO EC 18), yellow = ECs from the aorta at day 21 (AO EC 21), light green = SMCs from the aorta at day 18 (AO SMC 18), dark green = SMCs from the aorta at day 21 (AO SMC 21), turquoise = ECs from the DA at day 18 (DA EC 18), blue = ECs from the DA at day 21 (DA EC 21), purple = SMCs from the DA at day 18 (DA SMC 18), pink = SMCs from the DA at day 21).

**Figure 5:**
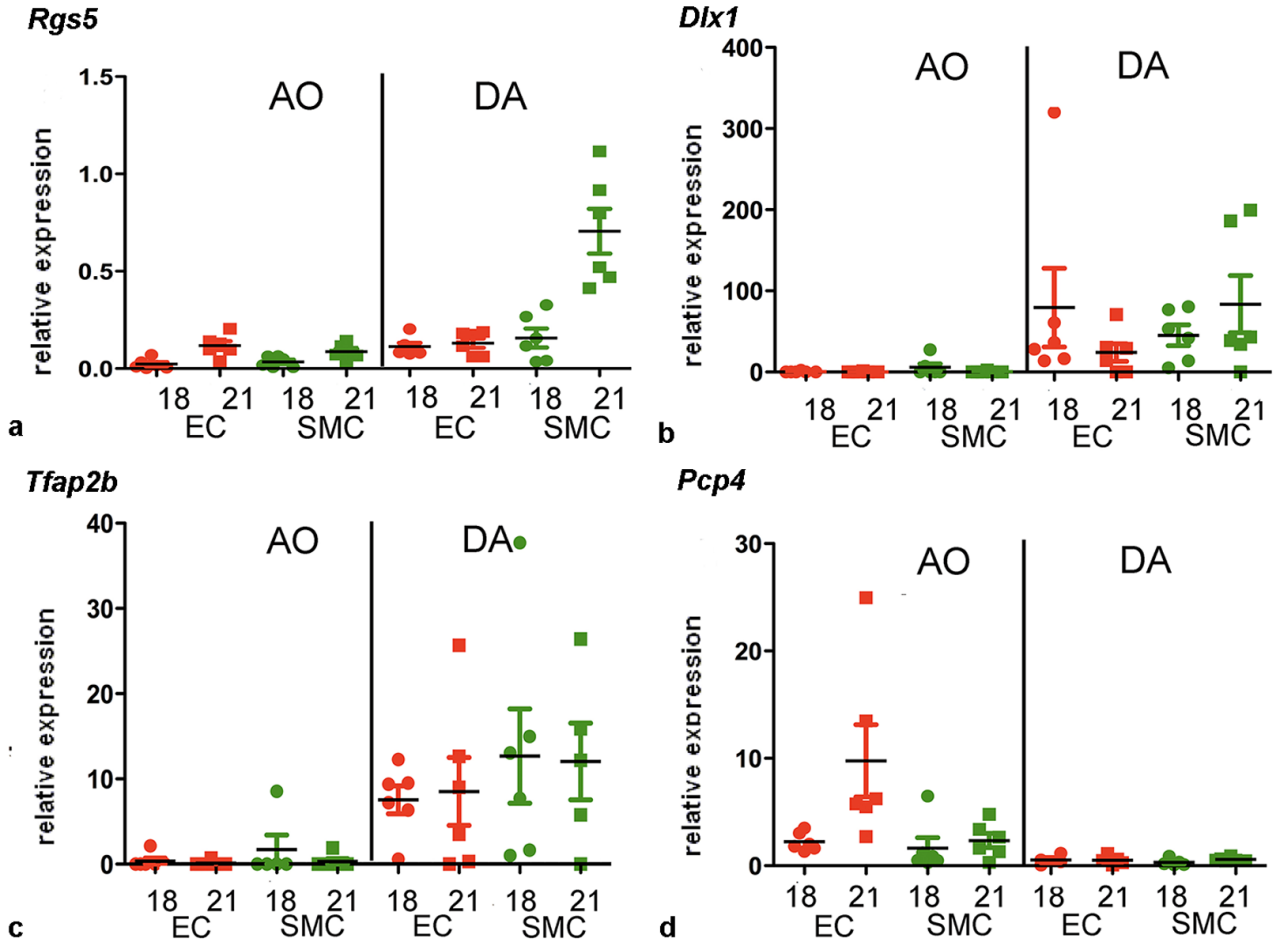
Gene expression of *Rgs5* (a), *Dlx1* (b), *Tfap2B* (c) and *Pcp4* (d) by rtqPCR. The same mRNA preparations were used for microarray (shown in figure 4) and rtqPCR. Relative expression levels are shown for each sample. Horizontal bars depict the means. The levels are peptidylprolylisomerase B (*Ppib*) normalized. Red symbols represent endothelial cells (EC) and green symbols represent smooth muscle cells (SMC). AO = aorta, DA = ductus arteriosus. 18 = day 18, 21 = day 21.

*Rgs5* was identified as a DA-enriched gene by the microarray. LIMMA revealed a significant upregulation of *Rgs5* in all four cell types of the DA at both time points (Figure 4a) showing higher expression levels at day 21 as compared to day 18. To confirm the findings we performed rtqPCR analyses (Figure 5a). Using a validated fluorogenic TaqMan® gene expression array (*Ppib normalized)* we confirmed the upregulation of *Rgs5* in the DA in comparison to the aorta. The highest relative expression of *Rgs5* was found in the SMCs of the DA at day 21. At day 18 RGS5 was significantly less expressed in the SMC of the DA confirming also the strong age-dependency of this gene in the DA. ECs of the DA expressed comparably low levels of *Rgs5* at day 18 and 21. In the aorta *Rgs5* was below the detection level in both cell types at day 18 and remained at a very low level at day 21.

### Dlx1

*Dlx1* was detected as a DA-enriched gene by the microarray. Globally *Dlx1* exhibited a 1.75 log-fold upregulation in the cells of the DA when compared to the cells from the aorta (Figure 4b). The highest expression levels of *Dlx1* were found in SMCs of the DA (Figure 4b). The upregulation in the DA was significant in both cell types at both time points. RtqPCR analysis (*Ppib* normalized) confirmed the upregulation of Dlx1 expression in the DA (Figure 5b). The clear expression difference was present in all cell types. *Dlx1* was virtually absent in aortic ECs of both gestational ages and in aortic SMCs of day 21 (0.3–0.4 fold-difference). In the DA at day 18 and day 21 both ECs and SMCs had a high magnitude of *Dlx1* expression (ranging between 24 and 84 fold-difference). An increase of the expression levels with gestational age was present in the SMCs but not detected in ECs.

### Tfap2B

*Tfap2B* was selected because of its differential expression between DA and aorta shown in the volcano-plot (Figure 4c). An upregulation of *Tfap2B* was documented in both cell types of the DA at both time-points (Figure 4c) showing higher expression levels at day 21 as compared to day 18. Using a validated fluorogenic TaqMan® gene expression array (*Ppib* normalized), we confirmed the ductus-specific expression in ECs en SMCs at day 18 and day 21 (Figure 5c). Differences between the expression levels in the SMC and the EC of the DA were not significant. In cells from the aorta *Tfap2B* expression was below or just slightly above detection level.

### Pcp4

*Pcp4* was recognized as an aorta-enriched gene with the highest and most significant level of differential expression between aorta and DA by LIMMA in ECs and SMCs at both time points (Figure 4d). Furthermore, the expression level of *Pcp4* was higher in all samples from day 21 as compared to day 18. LIMMA analysis also revealed the significant upregulation in the aorta (log-fold 3.2, adj. *P*<0.001) globally and also separately for gestational age and cell type. Because of its high and significant upregulation in the aorta, we selected *Pcp4* for confirmatory rtqPCR. The validated fluorogenic TaqMan® gene expression array *(Ppib* normalized) also showed an upregulation of this gene in the aorta compared to the DA (Figure 5d). The highest and most significant upregulation (10 fold-difference) was found in the ECs of the aorta at day 21 while the magnitude of expression was only 2 fold increased in aortic ECs at day 18. SMCs of the aorta did not show an increase of *Pcp4* expression with gestational age. In all samples of DA cells relative *Pcp4* expression was detected at very low levels (0.3–0.7 fold difference) confirming the microarray data.

## Discussion

DA maturation and closure is regulated by the interaction of many genes in different cell types. We performed a genome-wide microarray analysis searching for genes that are differentially expressed between DA and aorta of fetal rats at day 18 and day 21. Laser-capture microdissection was used to isolate ECs and SMCs as an efficient and precise method for the sampling of single cells or subgroups of cells in heterogeneous tissues. [24] This selective approach minimizes the noise of the microarray introduced by the variation in tissue composition. [25] We selectively amplified mRNA from ECs and SMCs from DA and aorta and intentionally did not pool the various samples. Analysis of the microarray data revealed for the most part DA- and aorta-enriched genes that were not detected in former studies [19], [20] in which microarrays of the complete wall of the aorta were performed. Among 16 differentially expressed genes between DA and aorta detected by LIMMA, this study for the first time identified *Rgs5* and *Dlx1* as molecular markers of the DA in the fetus. Together with *Pcp4*, the most significant aorta-enriched gene in our analysis, and *Tfap2B* these genes were studied more extensively with other techniques. In all analyses a significant time-dependency for *Rgs5*, *Dlx1* and *Pcp4* was observed. This strengthens the initial observation that gestational age had the largest influence on the differential gene expression between all samples.

The small overlap in detected genes between the current study and former studies is not surprising as differences are unavoidable when comparing transcriptional profiles by the microarray technique as Coceani et al. [21] emphasized commenting on the large variance between two former studies. [19],[20] Therefore it was suggested that any such comparison should consider cohorts of genes for distinct functions rather than single genes. [21] The current selective microarray study enables us to discuss single genes that were most significantly differentially expressed between ECs and SMCs in DA and aorta.

*Rgs5* is enriched in ECs and SMCs of the DA with a maturation–state dependent increase in expressionlevelbetweenday18 to day 21. *Rgs5* is coding for a protein regulating the function of vasoactive G-protein coupled receptors (GPCRs). [26] Many GPCRs are active during fetal maturation and postnatal closure of the DA such as EP2, 3, 4 [27], angiotensin [19] and ET-1 receptors. [28] *Rgs5* is the most highly and differently expressed *Rgs-R4* subfamily member in arterial smooth muscle, suggesting that *Rgs5* is a candidate for regulating arterial contractility,[29] probably under control of platelet-derived growth factor (PDGF) that represses RGS5. [26] *Rgs5* is downregulated in pericytes and vascular smooth muscle cells of *Pdgf* null mice. [30] The maturation–dependent upregulation of this DA-enriched gene in the newborn was not categorized in the fetal cohort. [19] Recently it was shown that the homogeneous Rgs5 expression in the aorta of neonatal mice changed into a mosaic pattern in adult animals. [31] The lowest expression levels were found in the ascending aorta and carotid arteries. Interestingly a localized high *Rgs5* expression was documented at the site of the former DA insertion [31] suggesting that an origin-specific epigenetic program resulting in differential *Rgs5* promoter methylation is involved in the regulation of *Rgs5* expression. The DA SMCs derive from neural crest cells populating the 6th pharyngeal arch, while the SMCs from the descending aorta are derived from the adjacent somites. We did not study the role of epigenetic factors causing differential *Rgs5* promoter methylation as shown in mice [31].

Microarray analysis also revealed *Dlx1* as a DA-enriched gene. It discriminates the DA from the aorta at day 18 and day 21 and is highly enriched in both ECs and SMCs of the DA as documented by quantitative rtPCR. This is a novel finding, Costa et al. [19] and Jin et al. [20] did not identify upregulation of *Dlx1* in the DA. Dlx1 is part of a subfamily of vertebrate homeobox-containing genes that are structurally similar to the *Drosophila* distal-less gene. *Dlx1* plays a role in the development of pharyngeal arch elements including jaws and teeth. Furthermore it is active during neurogenesis in the forebrain. [32] In addition Dlx genes have been detected in several cancer cell lines [33] where deregulation of Dlx genes was related with tumor progression. [33] *Dlx1* and *2* are expressed in the embryonic caudal pharyngeal arch complex where the ductus arteriosus develops. [34] This is also the case for two other homeobox gene families (*Msx* and *Prx]* that interact with *Dlx*. [35] Interestingly, *Prx2* is enriched in developing chicken DA while *Prx1* is enriched in the adjoining vessel parts. [36] The loss of function of both genes in gene-targeted mice results in DA malformations. [37] The interactions between *Msx*, *Prx2* and *Dlx1* genes in DA development have yet to be elucidated. In addition to its role in embryogenesis *DLX1* has been reported in hematopoietic cells [38] as a regulator of multiple signals from TGF-beta superfamily members. [39] *DLX2* the counterpart of *DLX1* in the bigene cluster is expressed depending on reactive oxygen species (ROS) in response to glucose deprivation. [40] Based on our data and the information from other biological contexts we propose that Dlx1 is involved in late gestational vascular remodeling of the DA.

Because of the significant upregulation of *Tfap2B* in the DA in our microarray experiment we selected this gene for further validation by rtqPCR. The AP-2B transcription factor is involved in the regulation of SMC development in the DA in mice. [17] Acting in a transcriptional network with ET-1 and HIF2a *Tfap2B* is shown to be relevant for proper ET-1 signaling in the DA. [17] Therefore it has been suggested that patent DA in *Tfap2B* deleted animals could partly be attributed to the lack of ET-1 signaling. Recently Zhao et al. [18] described the phenotype of *Tfap2B* knockout mice and documented Bmp2 and Bmp4 as downstream targets of Tfap2B. These mice are characterized by a PDA, postaxial accessory digits and enhanced apoptotic cell death of renal epithelial cells. [18], [41] Interestingly, *DLX1* directs the expression of *BMP4* in various biological contexts. [39] The upregulation of both transcription factor genes *Dlx1* and *Tfap2 B* in the DA suggests a vessel-specific regulation of *Bmp4* signaling during fetal DA remodeling.

*Pcp4* was identified as the most significant aorta-enriched gene. From day 18 to day 21 an increase in *Pcp4* levels was observed suggesting a biological function of *Pcp4* in the aorta during this period. However, the role of *Pcp4* and its protein has not been characterized sufficiently in the vascular system. Pep-19/pcp4-null mice have been made. [42] Although these mice are viable and fertile it cannot be ruled out that the maturation of the ductus arteriosus is impaired. In humans and also in rats [8] a small DA can persist with few haemodynamic consequences. Lacking a more detailed description of the vascular system and the litter sizes [42], perinatal mortality in these mice due to abnormal maturation of the DA or aorta might have been missed.

*Pcp4* has mainly been studied in neuronal tissues where it regulates calcium/calmodulin interactions [43] and inhibits calcium induced neuronal cell death by apoptosis. [44] By analogy to the neuronal tissues we postulate that upregulation of *Pcp4* in the aorta might protect the aorta around birth against apoptosis. Accordingly, the relatively lower expression of *Pcp4* in the DA might render the EC and SMC in the DA more susceptible to apoptosis, which plays an important role in postnatal DA closure. [5] Interestingly, a study on the parturition of mice linked another apoptosis driven process – the softening of the cervix at the end of gestation - with a relative decline in *Pcp4* expression in cervix tissue. [45] This supports a role for *Pcp4* as anti-apoptotic regulator. The results of Jin et al. [20] are in concordance with our results and categorized *Pcp4* as aorta-enriched gene in the fetus. Costa et al. [19] detected a postnatal upregulation of the same gene in the DA in comparison to the aorta. Additionally a cluster of other calcium-linked genes was identified in the neonatal DA, while its postnatal upregulation was related to a modified calcium homeostasis and increased drive of the DA to contraction. [19] Our analysis of mRNA from ECs and SMCs from fetal DA did neither identify Pcp4 nor one of the other members of the cluster of calcium-linked genes described in the neonatal DA.

In conclusion, our study confirms a DA-specific transcriptional profile in ECs and SMCs and thereby offers a basis to unravel the molecular regulation of fetal DA maturation. For the first time we recognized *Rgs5* and *Dlx1* as DA-enriched genes. These genes represent novel molecular targets for the regulation of fetal DA maturation and postnatal DA closure.

## Supporting Information

Figure S1 **Visual representation of microarray results of the pilot experiment.** **A:** Spectral map bioplot of samples of day 21 in the independent pilot experiment. The first principal components (PC) of the weighted spectral map analysis (SPM) of normalized microarray data are plotted. Colored squares with numbers depict different samples, while circles depict genes. Distances between the squares are a measure for similarity between samples. Genes that do not contribute to the differences between the samples are indicated as dots in the cloud around the centroid (represented by the cross). The ten most significantly contributing genes to the differences between samples are positioned in the largest distance from the centroid and annotated by their gene symbol. PC1 represented on the x-axis explains 28% of the variance of the dataset and discriminates between DA and aorta. PC2 represents 13% of the variance and discriminates between ECs and SMCs. Note *Vwf* among the genes that contribute to this difference. **B:** Volcano plot of the pilot experiment. Differential expression between samples of SMC of the DA and the aorta at day 21. The volcano plot constructed with LIMMA analysis summarizes the fold changes between the two types of samples (*i.e.,* DA versus aorta) and the log 10 transformed p-values. The negative log 10 transformed p-values (y-axis) are plotted against the log ratios between the samples (log_2_ fold change). From the four selected genes of our study *Dlx1* and *Rgs5* are found in the upper left of the plot. **C:** Volcano plot of the pilot experiment. Differential expression between EC of the DA and aorta. *Dlx1* (upper left) and *Pcp4* (upper right) both show a high ratio of differential expression in combination with a high significance level.
(TIF)

Figure S2 **Gene expression of** ***Rgs5*** **,** ***Dlx1, Pcp4*** **, and** ***Tcfap2B*** **at day 21 (pilot experiment).** Gene expression of *Rgs5 (* ***a*** *)*, *Dlx1 (* ***b*** *), Pcp4 (* ***c*** *)*, and *Tcfap2B (* ***d*** *)* at day 21 by microarray in the independent pilot experiment using 4 DA and 4 aorta samples. One DA sample was excluded because of mRNA degradation. Expression levels are expressed as fluorescent signal intensity measured on the array after normalization. Expression levels are shown for individual samples. The colors are the same as in figure S1 The colored horizontal lines represent the means.
(TIF)

Figure S3 **Protein expression of RGS5, DLX1 and PCP4 by immunohistochemistry.** Photomicrographs of representative transverse sections show (a,b) RGS5 (c,d) DLX1 and (e,f) PCP4 expression in DA and aorta. The DA (a) shows a more intense cytoplasmatic staining against RGS5 in ECs and SMCs than the aorta (b). This is most clearly seen in the EC and the subendothelial layer of SMCs which were also studied in the microarray experiment. Dlx1 is predominantely expressed in the DA (c) where EC and the innermost layers of SMC show the most intense staining. The EC in the aorta (d) are almost negative for DLX1. PCP4 is predominantely expressed in the aorta (f) the DA (e) shows less stained nuclei of the ECs of the aorta. In comparison with the aorta (f) the DA (e) shows less stained nuclei and cytoplasm of ECs and SMCs. Scale bars 100 µm.
(DOCM)

Figure S4 ***Vwf*** **gene expression results.** Gene expression of von Willebrand Factor (*Vwf)* by microarray and RT-PCR. Expression levels are expressed as fluorescent signal intensity measured on the array after normalization (**a**). Expression levels are shown for individual samples. The colors correspond to the colors used in figure 1. The colored horizontal lines represent the means. Black dots represent samples that are not reliably detected. Note the high expression level of *Vwf* in all EC samples. The graph (**b**) shows the relative quantification of mRNA of *Vwf* by quantitative RT-PCR (qRT-PCR) normalized with PGK1. The red symbols represent EC, the green SMC. All individual samples are presented. The horizontal lines indicate the means. The same mRNA preparations were used for microarray and the qRT-PCR. The PCR results confirm the microarray results by showing a high relative expression of *Vwf* in EC while its mRNA is just above detection level in SMC of the aorta.
(TIF)

Figure S5 **Separate volcano plots for each cell type and gestational age.** Volcano plots constructed with LIMMA analysis plotted separately for (**a**) EC at day 18, (**b**) SMC **at day 18, (c) EC at day** 21 and (**d**) SMC at day 21. The four selected genes *Rgs5*, *Dlx1*, *Tcfap2B* and *Pcp4* are all identified with high ratios of differential expression in combination with a high significance level in (**a**) and partly in (**b,c,d**).
(TIF)

Table S1 **Two-round linear mRNA amplification yield.** RNA yields of amplified RNA (aRNA) and biotin-labeled antisense mRNA (cRNA). Chip ID = Chip identification number, Individual samples are described by a four-digit number followed by a capital letter, sample site, cell type and gestational age. Aorta = descending aorta, DA =  ductus arteriosus, EC = endothelial cells, SMC = smooth muscle cells, 18 d = 18 days, 21 d = 21 d.
(DOC)

Table S2 **RtqPCR assay details.** This table contains the parameters derived by linear and robust regression of the pre-designed assays (Applied Biosystems) that were used for quantitative rt-PCR. The calibration curves obtained by robust regression are also represented in this supplementary table. All validated Applied Biosystems assays for *Dlx1* will detect sequences 5′ upstream of the 3′ sequence detected by the Affymetrix microarray probes. Therefore, specific primers/probe against this 3′ sequence were designed.
(XLS)
