# Supplementary figures and images for: Dlx1 and Rgs5 in the Ductus Arteriosus: Vessel-Specific Genes Identified by Transcriptional Profiling of Laser-Capture Microdissected Endothelial and Smooth Muscle Cells
Source: PLoS One. 2014 Jan 28;9(1):e86892. doi: 10.1371/journal.pone.0086892 (PMC3904938; doi:10.1371/journal.pone.0086892)

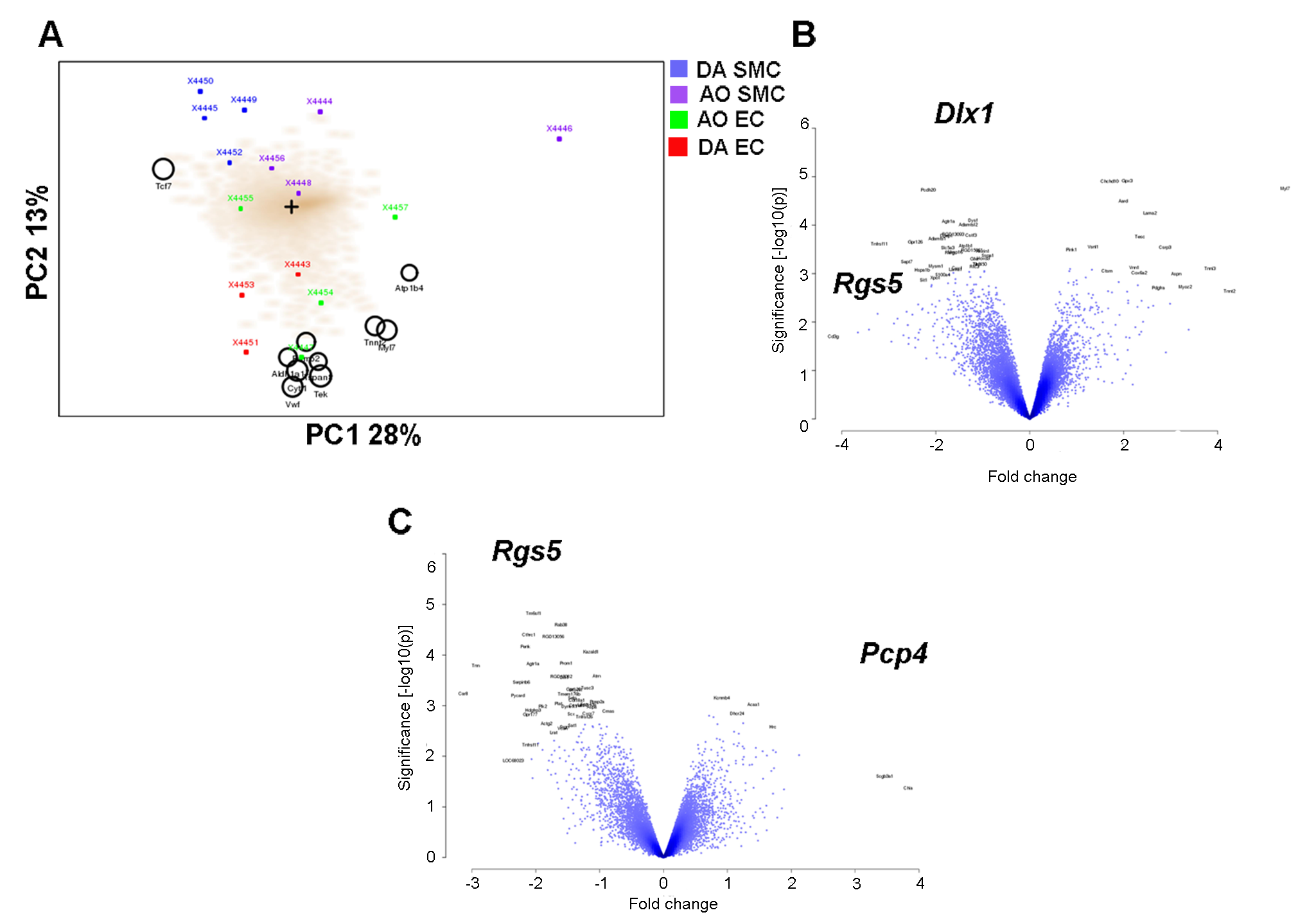

Supplement: Figure S1 — Visual representation of microarray results of the pilot experiment. A: Spectral map bioplot of samples of day 21 in the independent pilot experiment. The first principal components (PC) of the weighted spectral map analysis (SPM) of normalized microarray data are plotted. Colored squares with numbers depict different samples, while circles depict genes. Distances between the squares are a measure for similarity between samples. Genes that do not contribute to the differences between the samples are indicated as dots in the cloud around the centroid (represented by the cross). The ten most significantly contributing genes to the differences between samples are positioned in the largest distance from the centroid and annotated by their gene symbol. PC1 represented on the x-axis explains 28% of the variance of the dataset and discriminates between DA and aorta. PC2 represents 13% of the variance and discriminates between ECs and SMCs. Note Vwf among the genes that contribute to this difference. B: Volcano plot of the pilot experiment. Differential expression between samples of SMC of the DA and the aorta at day 21. The volcano plot constructed with LIMMA analysis summarizes the fold changes between the two types of samples (i.e., DA versus aorta) and the log 10 transformed p-values. The negative log 10 transformed p-values (y-axis) are plotted against the log ratios between the samples (log2 fold change). From the four selected genes of our study Dlx1 and Rgs5 are found in the upper left of the plot. C: Volcano plot of the pilot experiment. Differential expression between EC of the DA and aorta. Dlx1 (upper left) and Pcp4 (upper right) both show a high ratio of differential expression in combination with a high significance level. (TIF) [file pone.0086892.s001.tif]

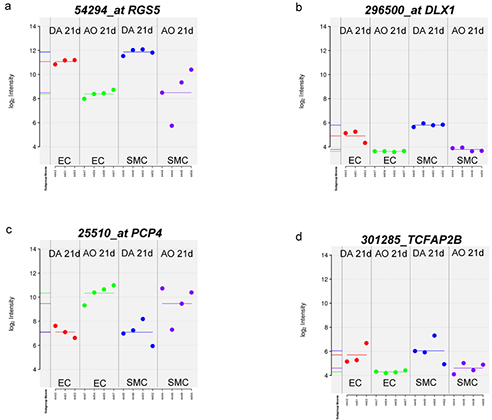

Supplement: Figure S2 — Gene expression of Rgs5 , Dlx1, Pcp4 , and Tcfap2B at day 21 (pilot experiment). Gene expression of Rgs5 ( a ), Dlx1 ( b ), Pcp4 ( c ), and Tcfap2B ( d ) at day 21 by microarray in the independent pilot experiment using 4 DA and 4 aorta samples. One DA sample was excluded because of mRNA degradation. Expression levels are expressed as fluorescent signal intensity measured on the array after normalization. Expression levels are shown for individual samples. The colors are the same as in figure S1 The colored horizontal lines represent the means. (TIF) [file pone.0086892.s002.tif]

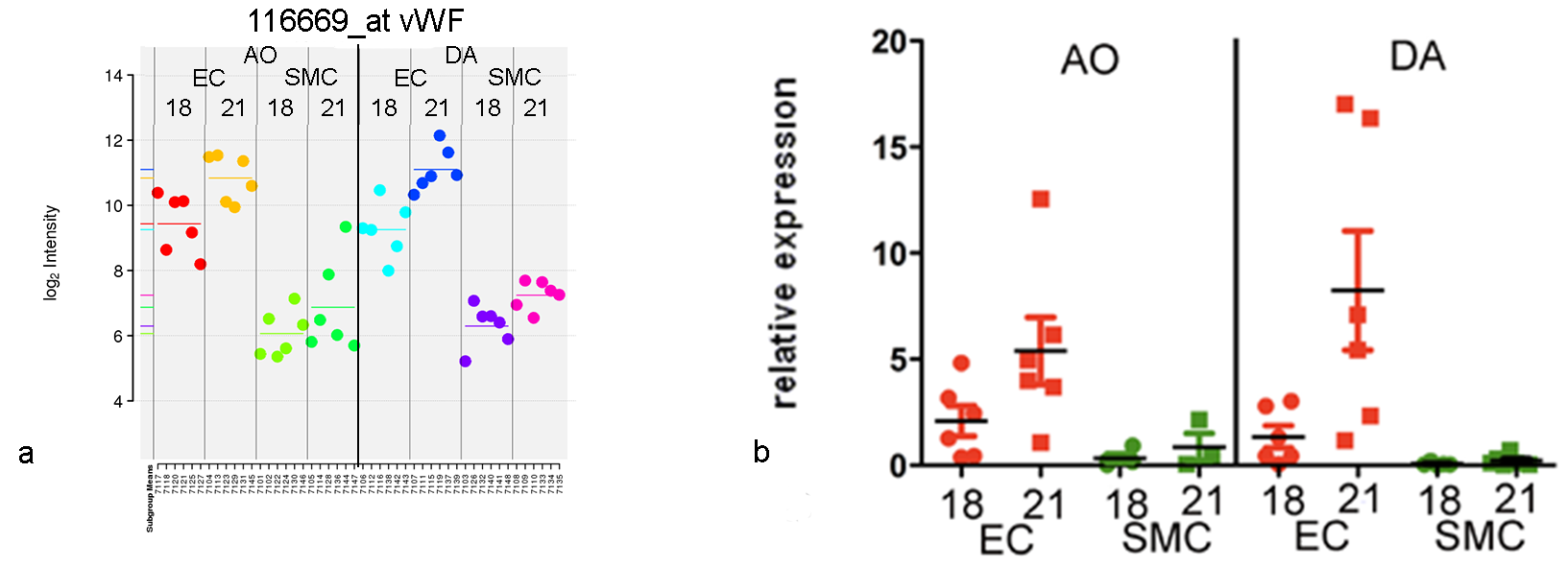

Supplement: Figure S4 — Vwf gene expression results. Gene expression of von Willebrand Factor (Vwf) by microarray and RT-PCR. Expression levels are expressed as fluorescent signal intensity measured on the array after normalization (a). Expression levels are shown for individual samples. The colors correspond to the colors used in figure 1. The colored horizontal lines represent the means. Black dots represent samples that are not reliably detected. Note the high expression level of Vwf in all EC samples. The graph (b) shows the relative quantification of mRNA of Vwf by quantitative RT-PCR (qRT-PCR) normalized with PGK1. The red symbols represent EC, the green SMC. All individual samples are presented. The horizontal lines indicate the means. The same mRNA preparations were used for microarray and the qRT-PCR. The PCR results confirm the microarray results by showing a high relative expression of Vwf in EC while its mRNA is just above detection level in SMC of the aorta. (TIF) [file pone.0086892.s004.tif]

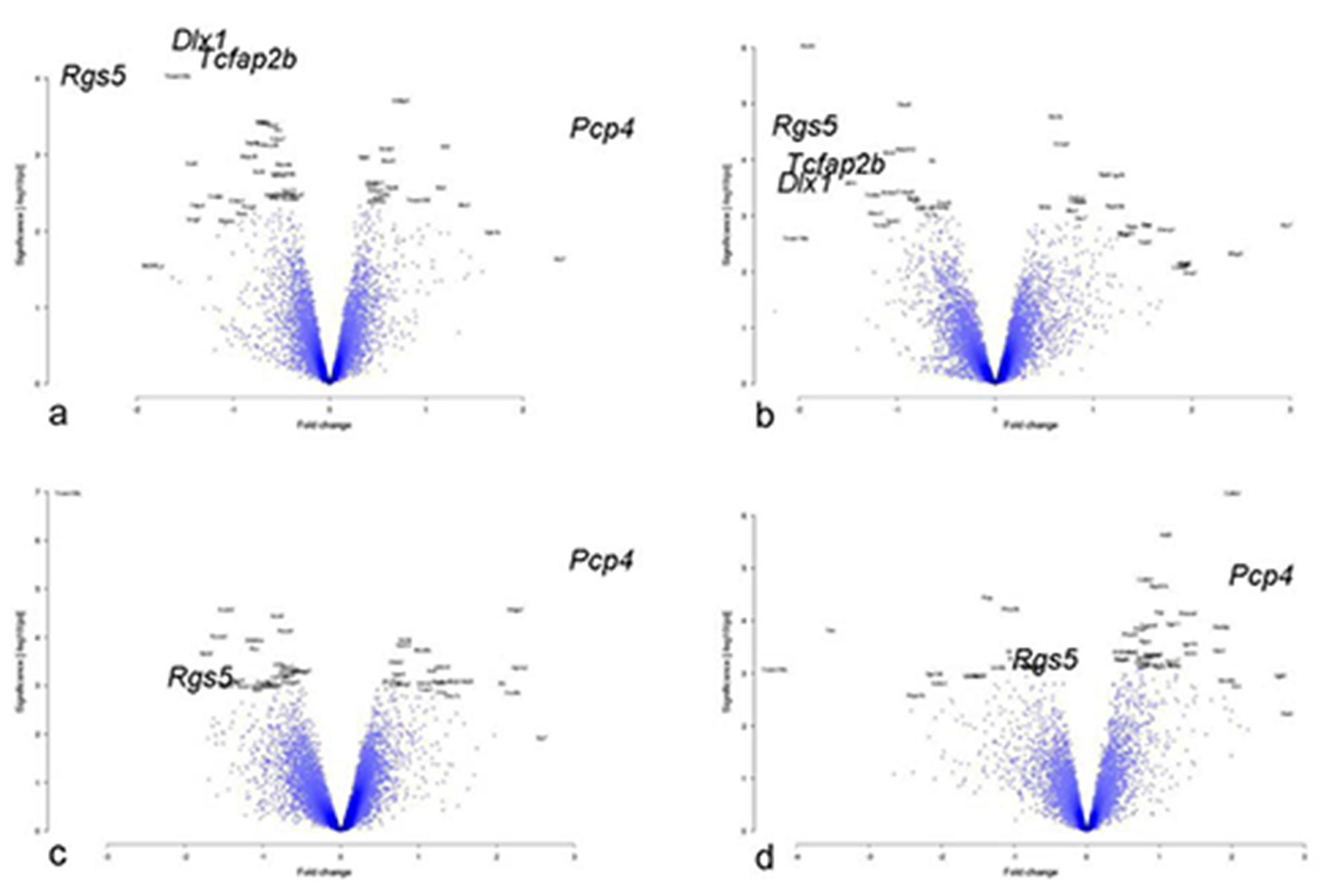

Supplement: Figure S5 — Separate volcano plots for each cell type and gestational age. Volcano plots constructed with LIMMA analysis plotted separately for (a) EC at day 18, (b) SMC at day 18, (c) EC at day 21 and (d) SMC at day 21. The four selected genes Rgs5, Dlx1, Tcfap2B and Pcp4 are all identified with high ratios of differential expression in combination with a high significance level in (a) and partly in (b,c,d). (TIF) [file pone.0086892.s005.tif]
