# Supplementary material for: Dlx1 and Rgs5 in the Ductus Arteriosus: Vessel-Specific Genes Identified by Transcriptional Profiling of Laser-Capture Microdissected Endothelial and Smooth Muscle Cells
Source: PLoS One. 2014 Jan 28;9(1):e86892. doi: 10.1371/journal.pone.0086892 (PMC3904938; doi:10.1371/journal.pone.0086892)
Supplement: Figure S3 — Protein expression of RGS5, DLX1 and PCP4 by immunohistochemistry. Photomicrographs of representative transverse sections show (a,b) RGS5 (c,d) DLX1 and (e,f) PCP4 expression in DA and aorta. The DA (a) shows a more intense cytoplasmatic staining against RGS5 in ECs and SMCs than the aorta (b). This is most clearly seen in the EC and the subendothelial layer of SMCs which were also studied in the microarray experiment. Dlx1 is predominantely expressed in the DA (c) where EC and the innermost layers of SMC show the most intense staining. The EC in the aorta (d) are almost negative for DLX1. PCP4 is predominantely expressed in the aorta (f) the DA (e) shows less stained nuclei of the ECs of the aorta. In comparison with the aorta (f) the DA (e) shows less stained nuclei and cytoplasm of ECs and SMCs. Scale bars 100 µm. (DOCM) [file pone.0086892.s003.docm]

**Immunohistochemistry**

The expression of Rgs5, Pcp4, and Dlx1 protein was visualized by immunohistochemistry in tissue samples of the DA and aorta. As the highest levels of mRNA expression of these genes were observed at day 21 we used this stage for the analysis of protein expression. Immunohistochemical staining revealed expression of Rgs5, Pcp4, and Dlx1 in all samples. Although immunohistochemistry is an essentially qualitative technique, we could confirm differences. The differential protein expression in DA and aorta confirmed the results of the microarray and rtqPCR. Rgs5 was expressed in the cytoplasm of EC and SMC. The highest intensity of staining was detected in SMC of the DA localized in the subendothelial part of the intima. ECs and SMCs in other parts of the DA wall were also more intensely stained than the aortic ECs and SMCs in the descending aorta. DLX1 staining resulted in cytoplasmatic positivity in most of DA and aorta. The highest intensity of Dlx1 staining was found in the EC of the DA and as a whole the DA was more intensely stained than the descending aorta. The expression of Tfap2B protein was not identified by immunohistochemistry in the DA or aorta by absence of available selective antibodies. Sections stained against Pcp4 exhibited a strong nuclear positivity and a weaker cytoplasmatic staining. The strong nuclear staining was most prominent in the EC of the aorta.

**Figure** **S5** Protein expression of Rgs5, Dlx1 and Pcp4 by immunohistochemistry. Photomicrographs of representative transverse sections show (a,b) Rgs5 (c,d) Dlx1 and (e,f) Pcp4 expression in DA and aorta. The DA (a) shows a more intense cytoplasmatic staining against Rgs5 in ECs and SMCs than the aorta (b). This is most clearly seen in the EC and the subendothelial layer of SMCs which were also studied in the microarray experiment. Dlx1 is predominantely expressed in the DA (c) where EC and the innermost layers of SMC show the most intense staining. The EC in the aorta (d) are almost negative for Dlx1. Pcp4 is predominantely expressed in the aorta (f) the DA (e) shows less stained nuclei of the Ec of the aorta. In comparison with the aorta (f) the DA (e) shows less stained nuclei and cytoplasm of ECs and SMCs. Scale bars 100 μm.
