# Supplementary material for: Dlx1 and Rgs5 in the Ductus Arteriosus: Vessel-Specific Genes Identified by Transcriptional Profiling of Laser-Capture Microdissected Endothelial and Smooth Muscle Cells
Source: PLoS One. 2014 Jan 28;9(1):e86892. doi: 10.1371/journal.pone.0086892 (PMC3904938; doi:10.1371/journal.pone.0086892)
Supplement: Table S1 — Two-round linear mRNA amplification yield. RNA yields of amplified RNA (aRNA) and biotin-labeled antisense mRNA (cRNA). Chip ID = Chip identification number, Individual samples are described by a four-digit number followed by a capital letter, sample site, cell type and gestational age. Aorta = descending aorta, DA = ductus arteriosus, EC = endothelial cells, SMC = smooth muscle cells, 18 d = 18 days, 21 d = 21 d. (DOC) [file pone.0086892.s006.doc]

| *Chip ID* | *Random no.* | *Sample* | *aRNA concentration ng/ml* | *aRNA*  *yield ng* | *cRNA*  *concentration ng/ml* | *cRNA*  *yield mg* | *cRNA concentration mg/ml* |
| --- | --- | --- | --- | --- | --- | --- | --- |
| 7101 | 1 | 8399 E Aorta SMC 18d | 24,69 | 493,8 | 2007,89 | 120,47 | 2,01 |
| 7102 | 2 | 8469 B Aorta SMC 18d | 11,01 | 220,2 | 2347,47 | 93,90 | 2,35 |
| 7103 | 3 | 8469 B DA SMC 18d | 13,44 | 268,8 | 1950,83 | 78,03 | 1,95 |
| 7104 | 4 | 8462 M Aorta EC 21d | 21,74 | 434,8 | 1601,12 | 64,04 | 1,60 |
| 7105 | 5 | 8462 M Aorta SMC 21d | 23,9 | 478 | 2558,39 | 102,34 | 2,56 |
| 7106 | 6 | 8469 F DA EC 18d | 19,87 | 397,4 | 1989,22 | 79,57 | 1,99 |
| 7107 | 7 | 8412 I DA EC 21d | 15,64 | 312,8 | 2462,99 | 98,52 | 2,46 |
| 7108 | 8 | 8412 I DA SMC 21d | 15,09 | 301,8 | 2633,82 | 105,35 | 2,63 |
| 7109 | 9 | 8462 A DA SMC 21d | 22,19 | 443,8 | 1677,63 | 67,11 | 1,68 |
| 7110 | 10 | 8462 C DA SMC 18d | 20,47 | 409,4 | 1149,93 | 46,00 | 1,15 |
| 7111 | 11 | 8462 A DA EC 21d | 26,47 | 529,4 | 1842,4 | 110,54 | 1,84 |
| 7112 | 12 | 8469 D DA EC 18d | 18,07 | 361,4 | 1480,23 | 59,21 | 1,48 |
| 7113 | 13 | 8412 I Aorta EC 21d | 24,9 | 498 | 802,44 | 32,10 | 0,80 |
| 7114 | 14 | 8412 I Aorta SMC 21d | 39,51 | 790,2 | 756,09 | 30,24 | 0,76 |
| 7115 | 15 | 8462 M DA EC 21d | 21,72 | 434,4 | 851,29 | 34,05 | 0,85 |
| 7116 | 16 | 8469 B DA EC 18d | 13,94 | 278,8 | 2894,31 | 115,77 | 2,89 |
| 7117 | 17 | 8399 H Aorta EC 18d | 25,61 | 512,2 | 2060,53 | 82,42 | 2,06 |
| 7118 | 18 | 8399 F Aorta EC 18d | 23,97 | 479,4 | 2323,01 | 92,92 | 2,32 |
| 7119 | 19 | 8412 D DA EC 21d | 21,7 | 434 | 1118,98 | 44,76 | 1,12 |
| 7120 | 20 | 8469 B Aorta EC 18d | 35,78 | 715,6 | 2078,61 | 83,14 | 2,08 |
| 7121 | 21 | 8399 E Aorta EC 18d | 15,19 | 303,8 | 1422,11 | 56,88 | 1,42 |
| 7122 | 22 | 8399 F Aorta SMC 18d | 18,95 | 379 | 1376,75 | 55,07 | 1,38 |
| 7123 | 23 | 8462 C Aorta EC 21d | 19,41 | 388,2 | 2273,48 | 90,94 | 2,27 |
| 7124 | 24 | 8469 D Aorta SMC 18d | 9,62 | 192,4 | 2184,54 | 87,38 | 2,18 |
| 7125 | 25 | 8469 F Aorta EC 18d | 32,07 | 641,4 | 1919,89 | 115,19 | 1,92 |
| 7126 | 26 | 8469 F DA SMC 18d | 25,8 | 516 | 1370,44 | 54,82 | 1,37 |
| 7127 | 27 | 8469 D Aorta EC 18d | 22,88 | 457,6 | 2054,34 | 82,17 | 2,05 |
| 7128 | 28 | 8412 A Aorta SMC 21d | 21,71 | 434,2 | 578,04 | 23,12 | 0,58 |
| 7129 | 29 | 8412 A Aorta EC 21d | 26,51 | 530,2 | 690,37 | 27,61 | 0,69 |
| 7130 | 30 | 8469 F Aorta SMC 18d | 17,82 | 356,4 | 1998,24 | 79,93 | 2,00 |
| 7131 | 31 | 8412 D Aorta EC 21d | 15,42 | 308,4 | 694,46 | 27,78 | 0,69 |
| 7132 | 32 | 8399 F DA SMC 18d | 27,22 | 544,4 | 1912,01 | 76,48 | 1,91 |
| 7133 | 33 | 8412 D DA SMC 21d | 19,43 | 388,6 | 769,08 | 30,76 | 0,77 |
| 7134 | 34 | 8462 M DA SMC 21d | 12,37 | 247,4 | 1649,3 | 65,97 | 1,65 |
| 7135 | 35 | 8412 A DA SMC 21d | 27,78 | 555,6 | 1688,72 | 67,55 | 1,69 |
| 7136 | 36 | 8412 D Aorta SMC 21d | 11,91 | 238,2 | 733,54 | 29,34 | 0,73 |
| 7137 | 37 | 8412 A DA EC 21d | 23,3 | 466 | 1181,48 | 47,26 | 1,18 |
| 7138 | 38 | 8399 F DA EC 18d | 28,29 | 565,8 | 1977,44 | 79,10 | 1,98 |
| 7139 | 39 | 8462 C DA EC 21d | 15,28 | 305,6 | 1607,9 | 64,32 | 1,61 |
| 7140 | 40 | 8399 H DA SMC 18d | 23,61 | 472,2 | 1787,44 | 107,25 | 1,79 |
| 7141 | 41 | 8469 B DA SMC 18d | 51,29 | 1025,8 | 1668,08 | 66,72 | 1,67 |
| 7142 | 42 | 8399 E DA EC 18d | 27,55 | 551 | 1998,44 | 119,91 | 2,00 |
| 7143 | 43 | 8399 H DA EC 18d | 39,05 | 781 | 2076,34 | 124,58 | 2,08 |
| 7144 | 44 | 8462 A Aorta SMC 21d | 23 | 460 | 2112,28 | 84,49 | 2,11 |
| 7145 | 45 | 8462 A Aorta EC 21d | 30,43 | 608,6 | 1278,07 | 51,12 | 1,28 |
| 7146 | 46 | 8399 H Aorta SMC 18d | 20,26 | 405,2 | 1528,84 | 61,15 | 1,53 |
| 7147 | 47 | 8462 C Aorta SMC 21d | 21,94 | 438,8 | 1654,4 | 66,18 | 1,65 |
| 7148 | 48 | 8399 E DA SMC 18d | 20,8 | 416 | 2694,16 | 107,77 | 2,69 |
